# Multimodal Hyperspectroscopy with LuViva^®^ for Detection of CIN2+ in Women Referred for Colposcopy: A Prospective Diagnostic Accuracy Study

**DOI:** 10.3390/cancers18203249

**Published:** 2026-10-09

**Authors:** Sonja Millert-Kalińska, Dominik Pruski, Marcin Przybylski

**Affiliations:** 1 Department of Oncological Gynecology, University Medical Hospital, Poznan University of Medical Sciences, ul. Szamarzewskiego 84, 60-569 Poznan, Poland; 2 Medicorner, Specialised Medical Practise, ul. Janusza Meissnera 2b/U3, 60-408 Poznan, Poland; 3 Gynecology and Obstetrics Clinical Hospital, Poznan University of Medical Sciences, ul. Polna 33, 60-535 Poznan, Poland

**Keywords:** LuViva, FRS—fluorescence and reflective spectroscopy, MHS—multimodal hyperspectroscopy, cervical triage, CIN2+, HSIL

## Abstract

**Objectives**: This study aims to evaluate the diagnostic performance of multimodal hyperspectral spectroscopy (MHS) using the LuViva^®^ Advanced Cervical Scan for the detection of cervical intraepithelial neoplasia grade 2 or worse (CIN2+) in women referred for further cervical assessment. **Methods**: This prospective, non-randomized diagnostic study included 77 women examined between September 2025 and April 2026. Participants were referred because of abnormal liquid-based cytology (LBC), high-risk human papillomavirus (HR-HPV) infection, or other indications for colposcopy. LuViva examination was performed before colposcopy with cervical biopsy. Histopathological diagnosis based on biopsy or loop electrosurgical excision procedure specimens was used as the reference standard. The sensitivity, specificity, positive predictive value, negative predictive value, and accuracy were calculated for HR-HPV testing, LBC, and different LuViva positivity thresholds. **Results**: The mean age was 39.0 ± 8.4 years. The final histopathology showed no lesions in 30 women, LSIL in 18, HSIL in 28, and cervical cancer in one. For CIN2+ detection, HR-HPV testing demonstrated a sensitivity of 93.1% and specificity of 29.2%, whereas LBC had a sensitivity of 82.8% and specificity of 39.6%. LuViva testing showed sensitivities between 82.8% and 100%, and a specificity—when comparing with LBC—of 45.8% but 20.8% in LuViva alone. LuViva results differ significantly between women with CIN2+ and those with LSIL or no lesions. **Conclusions**: LuViva showed potential as an adjunctive triage tool, as no CIN2+ lesions were observed among women with a low-risk result; however, the small number of negative tests and wide confidence intervals preclude safely deferring colposcopy. The AND strategy showed a modest numerical improvement in specificity and overall accuracy compared with LBC alone but did not show an increase in sensitivity; this finding should be considered exploratory.

## Simple Summary

Cervical cancer prevention increasingly relies on HPV testing, but a positive HPV result does not necessarily indicate a clinically significant cervical lesion. Therefore, additional methods are needed to better identify women who require further diagnostic procedures while avoiding unnecessary biopsies and colposcopies. In this prospective study, we evaluated LuViva^®^, a non-invasive device based on multimodal hyperspectral spectroscopy, in women referred for further cervical assessment because of abnormal screening results or other indications for colposcopy. LuViva showed high sensitivity for detecting high-grade cervical lesions, particularly when moderate- and high-risk results were considered positive, but its specificity was limited. Importantly, no high-grade lesions were observed among women with low-risk LuViva results in this study. These findings suggest that LuViva may have potential as an additional triage tool rather than as a standalone diagnostic test. However, larger studies are needed before it can be used to safely reduce unnecessary colposcopies or biopsies.

## 1. Introduction

Management of women with abnormal cervical cytology and high-risk human papillomavirus (HPV) subtypes remains a challenge. Given the increasingly widespread access to molecular diagnostics and the fact that patients are often younger, colposcopy with biopsy may sometimes be an excessive intervention. Consequently, clinicians continue to seek alternative methods characterized by high sensitivity, low invasiveness, and high patient acceptability. Infection with high-risk oncogenic HPV types is common among women aged 20–30, and the likelihood of the virus being cleared from cervical tissue reaches 90%; thus, while HR-HPV testing is sensitive, it lacks specificity for CIN2+ lesions. Poland is one of the few European countries where the incidence of cervical cancer remains high; according to data from the National Cancer Registry, approximately 2,400 women receive this diagnosis each year [1]. Although it is entirely detectable at early pre-cancerous stages and fully preventable, it continues to claim many lives. In Europe, the incidence of cervical cancer is 15 cases per 100,000 inhabitants, although there is a high variability between regions [2,3]. Moreover, clinicians are observing an increasingly alarming trend: due to earlier sexual initiation, changing relationship patterns, and the widespread use of oral contraceptives over barrier methods, CIN2+ lesions are being diagnosed in young women under the age of 30. Another noteworthy aspect is the absence of population-wide HPV vaccination in Poland. Although this preventive vaccine was added to the schedule of recommended vaccinations in 2006, it was initially available only on a commercial basis; the Universal HPV Vaccination Program was not launched until 2023. Initially, the program provided free vaccination to girls and boys aged 12 and 13, and the following year, the target group was expanded to include the 9–14 age range [4]. Consequently, in clinical practice, physicians observe two interesting groups: mature women who were not vaccinated against HPV in childhood and are now frequently HPV-positive, and young women who were vaccinated as children with the bivalent or quadrivalent vaccine and who initiated sexual activity at an early age.

Until 2025, Poland’s national cervical cancer prevention program relied on conventional Pap smears performed every three years [5]. According to the literature, the sensitivity of this test ranges from 45% to 60%; while it meets the criteria for a screening test, it is far from sufficient for detecting CIN2+ lesions. In July 2025, Poland introduced an alternative primary screening pathway based on HR-HPV testing with partial genotyping at five-year intervals. Reflex liquid-based cytology is performed using the same sample in women with a positive HR-HPV result. The national screening program currently covers women aged 25–64 years. This change resulted in significantly higher detection rates of HR-HPV positivity and a greater number of referrals for advanced cervical cancer diagnostics compared to previous practices. The most common genotypes in the Polish population are HPV 16 and 31; however, HPV 18 is not common [6,7,8]. The study authors therefore considered it worthwhile to perform an additional assessment using the LuViva device on a population referred for further diagnostic evaluation prior to the scheduled colposcopy and cervical biopsy. Similar studies have previously been conducted using different devices—TruScreen and Zedscan [9,10,11,12,13,14,15]. The aim of the current study was to evaluate the utility of fluorescence and reflectance spectroscopy—using the LuViva device—for the triage of patients indicated for colposcopy. The light beam reaches the basal layer of the epithelium—a layer not sampled during a cytology smear or molecular testing for HPV. Given the HPV virus’s affinity for the basal epithelial layer and its life cycle being closely linked to that of keratinocytes, collecting information from this layer is crucial [16,17,18,19].

The authors believe that if high sensitivity and specificity are confirmed, this approach could help reduce overdiagnosis and overtreatment—particularly in young, nulliparous patients—and limit the number of biopsies performed during colposcopy. A second aspect requiring long-term follow-up is the assessment of whether combining LBC results with LuViva findings can predict CIN2+ lesions.

## 2. Methods

### 2.1. Study Design

We conducted a prospective, non-randomized study in patients reporting to Medicorer, the Specialized Medical Practice in Poznan, Poland, between September 2025 and April 2026. Subjects attended the medical practice for in-depth diagnostic evaluation due to an abnormal LBC result or the presence of HPV 16, 18, or 31 in the cervical smear (according to the recommendations of the Polish Society of Obstetricians and Gynecologists). All patients requiring treatment of pre-cancerous HSIL lesions (CIN2+) underwent a loop electrosurgical excision procedure (LEEP) or a hysterectomy, in the case of cervical cancer, at the hospital. The Poznan University of Medical Sciences Bioethical Committee approved the study protocol (457/25) on 12 June 2025.

The inclusion criteria were as follows:

- Able to read or understand and give informed consent;
- Referral for LBC test within 120 days and/or HR-HPV test;
- Scheduled for colposcopy based on the 2025 Polish Cervical Cancer Prevention Program.

The exclusion criteria were as follows:

- Pregnancy;
- Menstruating on the day of colposcopy and LuViva test;
- Radiation therapy to her genitourinary system within 1 year;
- Prior hysterectomy in which the cervix was removed;
- Congenital anatomical cervical variant (e.g., double cervix);
- Post-coital or other significant bleeding at the time of the exam;
- Undergoing phototherapy;
- Recent use of photosensitizing agents, such as fluoroquinolones or retinoids.

The control group comprised adult women who voluntarily agreed to undergo cervical examination via the LuViva^®^ Advanced Cervical Scan. Only women with NILM LBC results and negative HR-HPV test results within 120 days were included.

### 2.2. The LuViva^®^ Advanced Cervical Scan (LuViva)

**Figure 1:**
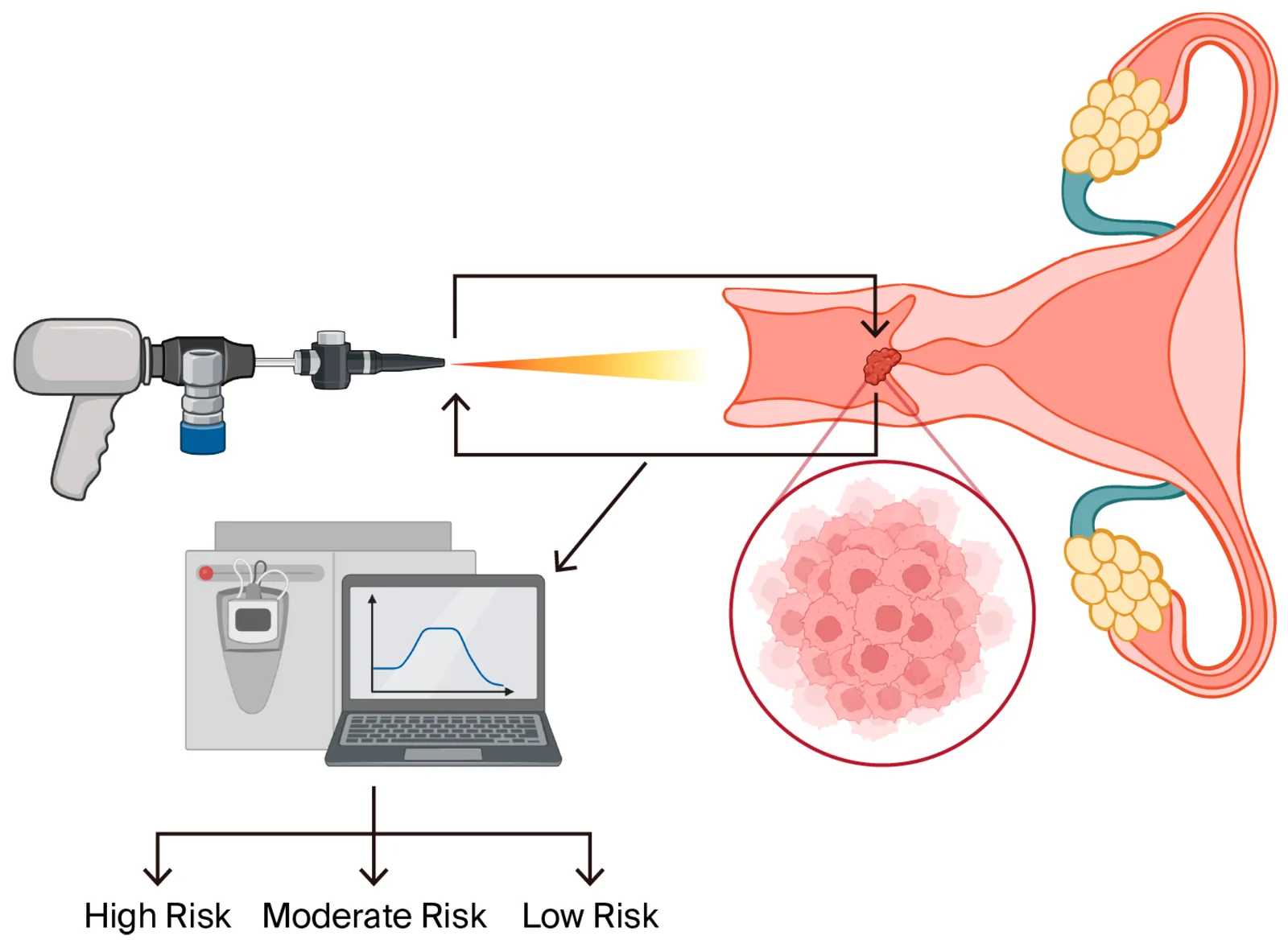
The examination scheme.

The LuViva^®^ Advanced Cervical Scan (LuViva) works by combining fluorescence and reflectance spectroscopy to identify chemical and physical changes in the uterine cervix. With the aid of the single-use LuViva^®^ Cervical Guide (CG), the device painlessly scans the cervix with light, analyzing the spectral data. After the scan, the results are displayed on the monitor for immediate review by the physician. To conduct a scan and receive results, there is no need to apply anything to the cervical tissue. Before examination, after placing a vaginal speculum, the cervix is cleared of any mucus, and then a CG is inserted. Following the prompts on the LuViva’s touchscreen monitor, the physician proceeds through the test steps until the completion of the scan, when the CG is removed. The LuViva’s results indicate the likelihood of CIN2+ within the scanned area as low, moderate, or high. The scheme of examination is shown in Figure 1.

For exploratory diagnostic accuracy analyses, LuViva results were dichotomized as positive when classified as moderate- or high-risk and negative when classified as low risk. This dichotomization was not prospectively specified as a clinical decision threshold and should therefore be regarded as exploratory. Combined LBC and LuViva performance was evaluated using two approaches: the AND strategy, in which both tests had to be positive, and the OR strategy, in which either test being positive was sufficient for an overall positive result.

### 2.3. Colposcopy, Punch Biopsy, and Cervical Treatment

Further validation of abnormal screening results was performed on all patients with an abnormal smear: ASC-US; LSIL; HSIL; ASC-H; AGC; cervical cancer; a positive HPV test for types 16, 18, and 31; and a clinically suspicious cervical image as described in a previous study. All patients with histopathologically confirmed CIN2+ underwent a LEEP or a hysterectomy in the case of cervical cancer.

### 2.4. Statistical Analysis

Statistical analyses were performed using R software (version 4.4.2). All hypothesis tests were two-sided, with a significance level of α = 0.05. Categorical variables were summarized as counts and percentages, whereas age was presented as mean and standard deviation (SD) due to its normal distribution. Normality was assessed using the Shapiro–Wilk test together with skewness and kurtosis measures. Comparisons between groups were performed using Student’s *t*-test (age) and Pearson’s chi-square test or Fisher’s exact test for categorical variables, as appropriate. The diagnostic performance of HPV testing, cytology, and LuViva classifications was evaluated using the histopathological outcome as the reference. The sensitivity, specificity, positive predictive value (PPV), negative predictive value (NPV), and accuracy were calculated and reported with 95% confidence intervals (CIs). Confidence intervals for diagnostic performance measures were calculated using the Wilson score method. All figures were created using https://BioRender.com.

## 3. Results

### 3.1. Characteristics of Total Study Group

The study included 77 women with a mean age of 39.0 ± 8.4 years, with a range of 23 to 57 years. HPV infection was detected in 79.2% of participants. The most frequent cytological diagnoses were NILM (31.2%), LSIL (27.3%), and ASC-US (20.8%). According to the final outcome, 39.0% of women had no lesions, 23.4% had LSIL, 36.4% had HSIL, and 1.3% had cancer (Table 1).

### 3.2. Comparison Between Patients with CIN2+ (HSIL + Cancer) in Final Histopathological Outcome and Other Patients (LSIL/Healthy)

Patients with CIN2+ (HSIL + cancer) in the histopathological outcome had a significantly higher proportion of positive HPV test outcomes compared to other patients (93.1% vs. 70.8%, *p* = 0.041). The detailed seven-category LBC outcomes did not reach statistical significance (*p* = 0.069, Supplementary Table S1), but after exploratory regrouping into NILM, ASC-US/LSIL, and ASC-H/HSIL/AGUS/CIS, a significant association with the final outcome was shown (*p* = 0.008). Among women with HSIL/cancer, 17.2% had NILM, 44.8% had ASC-US/LSIL, and 37.9% had ASC-H/HSIL/AGUS/CIS, compared with 39.6%, 50.0%, and 10.4%, respectively, among women with LSIL/no lesions. Biopsy HSIL+ findings were observed in 89.7% of women with a positive final outcome and in none of the remaining women. Similarly, the LEEP showed HSIL+ in 82.8% of women with a positive final outcome; however, it was not performed in 85.4% of women with LSIL/no lesions. Furthermore, a significant difference was observed in LuViva outcomes (*p* = 0.012). Low-risk outcomes fell significantly in the LSIL/no lesions group (20.8% of the LSIL/no lesions group). The proportion of moderate-risk and high-risk outcomes was higher in the HSIL/cancer group (13.8% vs. 6.2% and 86.2% vs. 72.9%, Table 2).

### 3.3. Diagnostic Performance of HPV, LBC, LuViva, and LBC Combined with LuViva in Predicting HSIL or Cancer

The diagnostic performance of HPV testing, LBC, and LuViva-based approaches for predicting HSIL/cancer in the final outcome is presented in Table 3. HPV testing demonstrated high sensitivity (93.1%, 95% CI: 78.0–98.1%) but low specificity (29.2%, 95% CI: 18.2–43.2%), resulting in an overall accuracy of 53.2% (95% CI: 42.2–64.0%). LBC alone demonstrated high sensitivity (82.8%, 95% CI: 65.5–92.4%) and substantially lower specificity (39.6%, 95% CI: 27.0–53.7%). The overall accuracy reached 55.8% (95% CI: 44.7–66.4%).

The results for LuViva combined with LBC differed from the predefined algorithm. The combination of LuViva and LBC using the “OR” criterion meant that one of the two results had to be abnormal—either the LBC result (ASC-US, LSIL, ASC-H, HSIL, AGUS, CIS) or the LuViva result—indicating moderate/high risk. The combination of LuViva and LBC using the “AND” criterion meant that both results had to be abnormal—the LBC result (ASC-US, LSIL, ASC-H, HSIL, AGUS, and CIS) and the LuViva result—indicating moderate/high risk. In Table 3, “LuViva+” is defined as a LuViva result indicating moderate/high risk.

**Figure 2:**
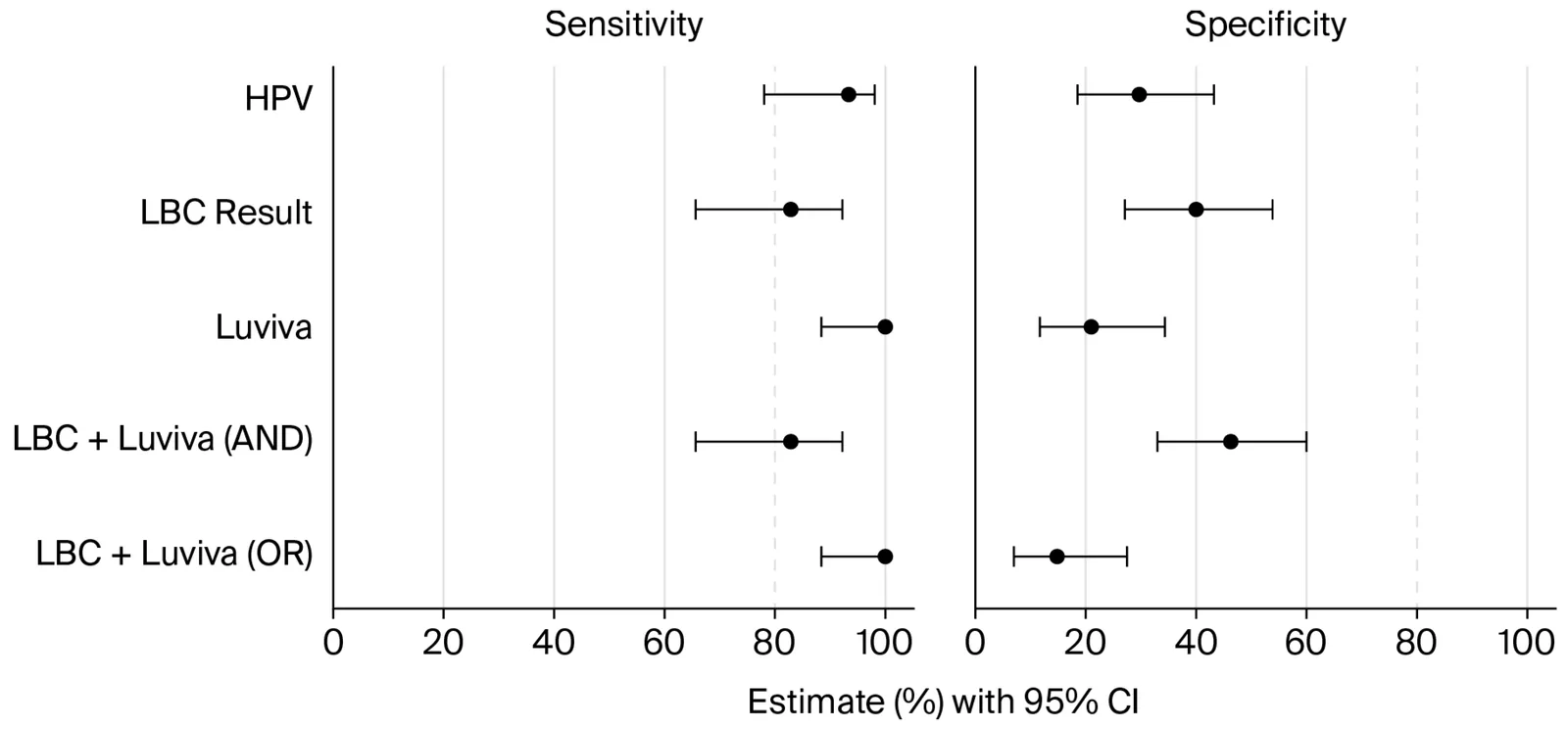
Diagnostic performance of HPV-, LBC-, and LuViva-based approaches for detection of positive final outcome defined as HSIL/cancer.

LuViva considered individually achieved a sensitivity of 100.0% (95% CI: 88.3–100.0%) and an NPV of 100.0% (95% CI: 72.2–100.0%), with a specificity of 20.8% (95% CI: 11.7–34.3%) and an overall accuracy of 50.6% (95% CI: 39.7–61.5%). LuViva combined with LBC using the AND criterion demonstrated a sensitivity of 82.8% (95% CI: 65.5–92.4%) and a specificity of 45.8% (95% CI: 32.6–59.7%), with an accuracy of 59.7% (95% CI: 48.6–70.0%). LuViva combined with LBC using the OR criterion achieved a sensitivity of 100.0% (95% CI: 88.3–100.0%) and an NPV of 100.0% (95% CI: 64.6–100.0%), accompanied by specificity of 14.6% (95% CI: 7.2–27.2%) and an accuracy of 46.8% (95% CI: 36.0–57.8%). All results are shown in Figure 2.

## 4. Discussion

The aim of this study was to evaluate a new adjunctive test performed prior to colposcopy; once refined, this test could potentially serve as a painless, objective alternative and a triage tool for patients with abnormal cytology results. The principal finding of this study was that a significant difference was observed in LuViva’s ability to predict CIN2+ lesions. None of the patients with a low-risk LuViva result received a final diagnosis of HSIL or cancer. LuViva testing identified all patients requiring further diagnostic workup and treatment involving an excision procedure; however, the number of cases was small, and each proposed management protocol requires validation in a prospective study. Among patients with HSIL, 86.2% had a high-risk LuViva result, while 13.8% had a moderate-risk result. However, it is essential to analyze the findings in the context of clinical data and, for the time being, consider it only as a potential application. This option does not interfere with established standards of care. Diagnostic tests utilizing artificial intelligence are gaining increasing popularity in gynecology as well [20,21]. When comparing our results to the largest study conducted in Europe to date—carried out by researchers from Hungary on a group of 208 women—certain similarities can be observed. According to Krasznai Zoárd Tibor et al. [22], cytology-based screening alone resulted in a high false-positive rate (69.28%). The false-positive rate of the spectroscopy examination (54.81%) was higher than that of the HPV testing (24.24%). In the detection of CIN2+, the spectroscopy had a 94% sensitivity (95% CI = 0.84–0.99), with a 22% specificity (95% CI = 0.15–0.31), a 90% negative predictive value (95% CI = 0.73–0.98), and a 34% positive predictive value (95% CI = 0.26–0.43) (*p* = 0.00130). According to our data, the NPV is 87.5% for HPV, 79.2% for LBC, and 100% for the LuViva device. When interpreting the combined LuViva and LBC result, the NPV was 81.5% (using the AND criterion).

Although the observed NPV of LuViva was 100%, this estimate was based on only 10 low-risk results and was associated with a wide 95% confidence interval (72.2–100%). Therefore, the apparent absence of CIN2+ among women with a low-risk LuViva result should be considered hypothesis-generating rather than evidence that a negative LuViva result can reliably exclude CIN2+. In particular, these findings do not currently support using a low-risk LuViva result as a basis for deferring colposcopy or biopsy. Prospective validation in a substantially larger cohort with a predefined positivity threshold is required before such a clinical strategy can be considered. The Hungarian study indicated that the PPV for MHS was lower than that of cytology and comparable to that of HPV testing. A similar numerical pattern was observed in our study based on the nominal PPV values. This means that the LuViva result should be interpreted with caution and not in isolation. The result should serve as a supplement; making diagnostic or therapeutic decisions without the results of other tests and clinical information is not recommended. A limitation of the Hungarian study is the lack of access to the full text of the publication in English and information regarding the type of cytology sample collected—conventional (slide-based) or liquid-based (thin-layer)—which makes the comparison between the studies somewhat flawed [22].

In 2020, a study by Turkish authors utilized the non-invasive nature of the examination to assess the risk of abnormalities in pregnant women [23]. Their study aimed to measure the performance of cervical multimodal hyperspectroscopy in comparison to Pap smears and HPV testing in low-risk pregnant women. The specificity of MHS in pregnant women with normal Pap smear results is estimated to be 92.9%. The procedure was also well-tolerated, with a patient satisfaction rate of 92%. These results cannot be compared with the data mentioned above, as the study population consisted primarily of women with normal cytology findings. Furthermore, the impact of pregnancy on the appearance of the cervix—specifically softening and decidual changes—must also be taken into account. On the other hand, the high rate of positive feedback from women regarding this test indicates that it is an interesting, non-invasive alternative to colposcopy with biopsy.

Interesting results were presented by Leo B. Twiggs et al. regarding 1607 women referred for colposcopy with biopsy due to abnormal screening results, 804 of whom also had two-year follow-up data available [24]. The sensitivity of MHS for cervical intraepithelial neoplasia (CIN) 2 + was 91.3% (252/276). The specificity, or the potential reduction in referrals to colposcopy and biopsy, was 38.9% for women with benign histology and 30.3% (182/601) for women with LSIL (CIN1) histology. Two-year follow-up data revealed 67 interval CIN2+ cases that originally were diagnosed at enrollment as normal or CIN1. MHS identified 60 of these (89.6%) as positive for CIN2 + prior to their discovery during the two-year follow-up period. These findings suggest that MHS may identify some lesions before they become apparent using conventional diagnostic methods; however, prospective external validation is required.

The main strengths of this study are its prospective design and the within-participant comparison of diagnostic methods, which allowed direct assessment of LuViva under the same clinical conditions. Histopathology was used as the reference standard.

Several limitations should be acknowledged. The relatively small sample size and the limited number of negative LuViva results reduced the precision of the diagnostic accuracy estimates and contributed to wide confidence intervals. The study population consisted of women referred for further diagnostic evaluation because of abnormal screening or clinical findings, which may have introduced selection and spectrum bias. Therefore, the findings may not be directly generalizable to women undergoing triage after a positive primary HPV screening test in population-based screening programs. Histological verification was based on either targeted biopsy or LEEP specimens, and differences in sampling volume may have affected lesion detection and grading. Non-uniform verification of the reference standard may also have introduced partial/differential verification bias. Because women with negative or CIN1 biopsy findings were not routinely subjected to excisional verification, occult or unsampled CIN2+ lesions cannot be completely excluded in this group, which could have resulted in an overestimation of sensitivity and NPV. Finally, longitudinal follow-up was not incorporated into the reference standard for the present diagnostic-accuracy analysis, precluding assessment of the relationship between LuViva results and subsequent lesion regression, persistence, or progression. Larger prospective studies with standardized verification and longitudinal follow-up are needed to further define the role of LuViva in HPV-based cervical cancer screening and triage.

## 5. Conclusions

In this prospective study, multimodal hyperspectroscopy demonstrated that no CIN2+ lesions were observed among women with a low-risk LuViva result in this cohort; however, the low number of negative tests and the wide confidence interval mean that this finding is not sufficient to safely defer colposcopy. It appears that the AND strategy showed the numerically highest overall accuracy among the evaluated LuViva–LBC combinations, mainly through a modest numerical improvement in specificity without further loss of sensitivity compared with LBC alone. Currently, given the small study group and the lack of definitive recommendations, this device cannot currently be recommended as a standalone triage method or as a basis for foregoing colposcopy or biopsy. However, it is promising and non-invasive, and can successfully be used as an additional test prior to colposcopy. The visual and immediate test result also offers educational value for patients. Further studies should evaluate predefined classification algorithms in larger, consecutively recruited populations with uniform histopathological verification.

## Figures and Tables

**Table 1:** Study group characteristics.

Characteristic	Statistic
*N*	77 (100.0)
Age, years, mean ± SD	39.03 ± 8.44
Study group	67 (87.0)
Control group	10 (13.0)
Cytology, *n* (%)	
NILM	24 (31.2)
ASC-US	16 (20.8)
LSIL	21 (27.3)
ASC-H	8 (10.4)
HSIL	6 (7.8)
AGUS	1 (1.3)
CIS	1 (1.3)
HPV, *n* (%)	
Positive	61 (79.2)
Negative	16 (20.8)
Biopsy, *n* (%)	
No lesions	30 (39.0)
LSIL	21 (27.3)
HSIL	24 (31.2)
SMILE	1 (1.3)
Cancer	1 (1.3)
LEEP, *n* (%)	
No lesions	5 (6.5)
LSIL	7 (9.1)
HSIL	23 (29.9)
Cancer	1 (1.3)
Not performed	41 (53.2)
Final outcome, *n* (%)	
No lesions	30 (39.0)
LSIL	18 (23.4)
HSIL	28 (36.4)
Cancer	1 (1.3)

SD, standard deviation.

**Table 2:** Comparison of patients with final CIN2+ histopathological outcome (HSIL/cancer) and other patients (LSIL/healthy).

Characteristic	Final Outcome		*p*
	HSIL/Cancer (*n* = 29)	LSIL/No Lesions (*n* = 48)	
Age, years, mean ± SD	37.21 ± 8.37	40.12 ± 8.37	0.143
LBC result, *n* (%)			
NILM	5 (17.2)	19 (39.6)	0.008
ASC-US/LSIL	13 (44.8)	24 (50.0)	
ASC-H/HSIL/AGUS/CIS	11 (37.9)	5 (10.4)	
HPV, *n* (%)			
Positive	27 (93.1)	34 (70.8)	0.041
Negative	2 (6.9)	14 (29.2)	
Biopsy, *n* (%)			
≤CIN1	3 (10.3)	48 (100.0)	-
HSIL+	26 (89.7)	0 (0.0)	
LEEP, *n* (%)			
≤CIN1	5 (17.2)	7 (14.6)	-
HSIL+	24 (82.8)	0 (0.0)	
Not performed	0 (0.0)	41 (85.4)	
LuViva outcome, *n* (%)			
Low	0 (0.0)	10 (20.8)	0.012
Moderate	4 (13.8)	3 (6.2)	
High	25 (86.2)	35 (72.9)	

SD, standard deviation. Comparisons were made with Student’s *t*-test (age), Pearson’s chi-square test (HPV positive/negative, LBC), or Fisher’s exact test (LuViva). LBC categories were grouped into NILM, ASC-US/LSIL, and ASC-H/HSIL/AGUS/CIS for exploratory analyses. Biopsy categories were grouped as follows: (1) ≤CIN1, including no lesions and LSIL outcomes, and (2) HSIL+, including HSIL, SMILE, and cancer. LEEP categories were grouped as follows: (1) ≤CIN1, including no lesions and LSIL outcomes, and (2) HSIL+, including HSIL and cancer. Statistical testing was not performed for biopsy and LEEP findings because those outcomes contributed directly to the definition of the final outcome.

**Table 3:** Diagnostic quality of test alternatives for predicting HSIL/cancer in final outcome.

Test		Final Outcome		Sensitivity, %	Specificity, %	PPV, %	NPV, %	Accuracy, %
		+	−					
HPV	+	27	34	93.1 (78.0–98.1)	29.2 (18.2–43.2)	44.3 (32.5–56.7)	87.5 (64.0–96.5)	53.2 (42.2–64.0)
	−	2	14					
LBC (ASC-US, LSIL, ASC-H, HSIL, AGUS, CIS)	+	24	29	82.8 (65.5–92.4)	39.6 (27.0–53.7)	45.3 (32.7–58.5)	79.2 (59.5–90.8)	55.8 (44.7–66.4)
	−	5	19					
LuViva (positive: high/moderate)	+	29	38	100.0 (88.3–100.0)	20.8 (11.7–34.3)	43.3 (32.1–55.2)	100.0 (72.2–100.0)	50.6 (39.7–61.5)
	−	0	10					
LBC + LuViva (LBC AND LuViva+)	+	24	26	82.8 (65.5–92.4)	45.8 (32.6–59.7)	48.0 (34.8–61.5)	81.5 (63.3–91.8)	59.7 (48.6–70.0)
	−	5	22					
LBC + LuViva (LBC OR LuViva+)	+	29	41	100.0 (88.3–100.0)	14.6 (7.2–27.2)	41.4 (30.6–53.1)	100.0 (64.6–100.0)	46.8 (36.0–57.8)
	−	0	7					

PPV, positive predictive value; NPV, negative predictive value. All measures are presented with 95% confidence intervals (CIs).

## Data Availability

Data are available upon request to the corresponding author.

## Supplementary Materials

The following supporting information can be downloaded at https://www.mdpi.com/article/10.3390/cancers18203249/s1, Table S1: Detailed outcomes of LBC, biopsy and LEEP in group with CIN2+ histopathological final outcome (HSIL/cancer) and other patients (LSIL/healthy).
