# Supplementary material for: Multimodal Hyperspectroscopy with LuViva® for Detection of CIN2+ in Women Referred for Colposcopy: A Prospective Diagnostic Accuracy Study
Source: Cancers (Basel). 2026 Oct 9;18(20):3249. doi: 10.3390/cancers18203249 (PMC13702515; doi:10.3390/cancers18203249)
Supplement: Supplementary file 1 [file cancers-18-03249-s001.zip › cancers-4561340-supplementary.pdf]

cancers

MDPI

**Supplementary Table S1.** Detailed outcomes of LBC, biopsy and LEEP in group with CIN2+ histopathological final outcome (HSIL/cancer) and other patients (LSIL/healthy).

| Characteristic | Final outcome | | *p* |
|---|---|---|---|
| | **HSIL/cancer (*n* = 29)** | **LSIL/no lesions (*n* = 48)** | |
| LBC result, *n* (%) | | | |
| NILM | 5 (17.2) | 19 (39.6) | 0.069 |
| ASC-US | 5 (17.2) | 11 (22.9) | |
| LSIL | 8 (27.6) | 13 (27.1) | |
| ASC-H | 5 (17.2) | 3 (6.2) | |
| HSIL | 4 (13.8) | 2 (4.2) | |
| AGUS | 1 (3.4) | 0 (0.0) | |
| CIS | 1 (3.4) | 0 (0.0) | |
| Biopsy, *n* (%) | | | |
| No lesions | 0 (0.0) | 30 (62.5) | - |
| LSIL | 3 (10.3) | 18 (37.5) | |
| HSIL | 24 (82.8) | 0 (0.0) | |
| SMILE | 1 (3.4) | 0 (0.0) | |
| Cancer | 1 (3.4) | 0 (0.0) | |
| LEEP, *n* (%) | | | |
| No lesions | 2 (6.9) | 3 (6.2) | - |
| LSIL | 3 (10.3) | 4 (8.3) | |
| HSIL | 23 (79.3) | 0 (0.0) | |
| Cancer | 1 (3.4) | 0 (0.0) | |
| Not performed | 0 (0.0) | 41 (85.4) | |

Comparison for LBC results made with Pearson's chi-square test.
